# Evolution, dissemination, and genetic dynamics of the carbapenem resistance gene *bla*_NDM_ in China

**DOI:** 10.3389/fcimb.2025.1608826

**Published:** 2025-08-11

**Authors:** Xiaofeng Hu, Boqian Wang, Mingliang Chen, Kexin Li, Zhixi Peng, Lianqun Jin, Junjie Yue, Hui Chen, Ling Zhang, Shaofu Qiu, Hongguang Ren, Hongbin Song

**Affiliations:** ^1^ Chinese People’s Liberation Army of China (PLA) Center for Disease Control and Prevention, Beijing, China; ^2^ Beijing Institute of Biotechnology, State Key Laboratory of Pathogen and Biosecurity, Beijing, China; ^3^ Institute of Pathology and Southwest Cancer Center, Southwest Hospital, Army Medical University, Chongqing, China; ^4^ Institute of Toxicology, School of Military Preventive Medicine, Army Medical University, Chongqing, China; ^5^ State Key Laboratory of Trauma, Burn and Combined Injury, Third Military Medical University, Chongqing, China; ^6^ Institute of Pathology and Southwest Cancer Center, China Medical University, Shenyang, China; ^7^ Department of Laboratory Medicine, 73 Army Hospital, Xiamen, China

**Keywords:** *bla*_NDM_, evolution, dissemination, *tet(X)*, genetic dynamics

## Abstract

**Background:**

*Bla*_NDM_, which encodes a metallo-β-lactamase that can hydrolyze most β-lactam antibiotics, has become a serious public health concern in China. It is crucial to investigate the evolution, dissemination, and genetic dynamics of *bla*_NDM_ to develop potential strategies to control the proliferation of *bla*_NDM_.

**Methods:**

In this study, we collected 1021 *bla*_NDM_-positive isolates, which features 67 new genomes from our laboratory and 954 genomes from NCBI. Through epidemiological big data analysis, phylogenetic tree-based geographic transmission analysis, and upstream-downstream genetic clustering evolution analysis, we systematically analyzed the evolution, dissemination, and genetic dynamics of *bla*_NDM_-positive bacteria.

**Results:**

Analysis results indicate that bla_NDM-5_ is gradually supplanting *bla*_NDM-1_ in China and Acinetobacter has been replaced as the primary *bla*_NDM_-harboring genus by the Enterobacter, Escherichia, and Klebsiella, which are both within the Enterobacteriaceae family and more easily transmitted among humans. Furthermore, *bla*_NDM_-positive bacteria exhibit a distinct livestock-environment-human transmission cycle, while the phylogenetic diversity of *bla*_NDM_ and *tet(X)*-co-carrying genera is progressively expanding with concomitantly enhanced resistance phenotypes. Currently, the predominant *bla*_NDM_-positive bacterial strains have likely disseminated from southwest China to coastal regions. We further identified multiple transposon structures beyond Tn125 that may facilitate *bla*_NDM_ transfer.

**Conclusions:**

The diversity of the *bla*_NDM_ and its carrier bacterial strains is continuously increasing, and its transmission range is also expanding. Of greater concern, super-resistant strains co-harboring *bla*_NDM_ and *tet(X)* genes exhibit high potential for imminent emergence in human populations. Considering that the *bla*_NDM_-carrier bacteria are increasingly adapted to inter-human spread, the analysis results above can provide methodological and data support for epidemiological surveillance, tracing, and early warning alerts.

## Introduction

Antimicrobial resistance can result from point mutations of non-antimicrobial resistance genes or horizontal transfer of antimicrobial resistance genes from other strains (Boolchandani et al., 2019). New Delhi metallo-β-lactamase gene is an antimicrobial resistance gene that encodes a metallo-β-lactamase capable of hydrolyzing most β-lactam antibiotics, which are often utilized to manage critical infections of multidrug-resistant Gram-negative bacteria. The spread of *bla*_NDM_-positive bacteria has received widespread attention due to the potential impact on human health (Khan et al., 2017; Usmanqamar et al., 2021).

*Bla*_NDM_ was initially identified in 2008 from a *Klebsiella pneumoniae* strain isolated from a urine sample from a patient in Sweden who had recently returned to the country from India (Yong et al., 2009). Although the worldwide spread the *bla*_NDM_ can be traced to India in most cases, where *bla*_NDM_ was first identified in 2005, an *bla*_NDM_-positive strain was isolated from a patient in Turkey with no history of travel outside of the country (Acman et al., 2022). Thus, accurate identification of the geographic origin of *bla*_NDM_ is challenging (Poirel et al., 2011; Ignasi et al., 2014). *Bla*_NDM_ predominantly occurs in the phylum Proteobacteria and has been confirmed in at least 11 different bacterial families (Fanny et al., 2020). *bla*_NDM_ is frequently encoded by plasmids but can also be carried on the chromosomes of some strains (Baraniak et al., 2016; Rahman et al., 2018). Generally, the genome of *bla*_NDM_-positive strains codes for only one *bla*_NDM_, although rare strains can harbor multiple *bla*_NDM_ genes, either within the same subtype or across different subtypes (Hu et al., 2017). The National Center for Biotechnology Information (NCBI) database has documented 32 *bla*_NDM_ subtypes, which are primarily the result of point mutations, with *bla*_NDM-1_ being the most prevalent subtype (Basu, 2020). From a broader perspective, *bla*_NDM_ is characterized by a range of genomic contexts based on the Tn125 backbone, which represents the most commonly occurring transposon structure (Wailan et al., 2015). Recently, several other transposon structures have been discovered involving various insertion sequence (IS) and transposase (Tn) elements related to *bla*_NDM_, including *ISAba125*, *IS5*, *IS6*, *IS26*, and *Tn3*, among others (Campos et al., 2015; Zhao et al., 2021; Zheng et al., 2021). Of these, *ISCR27* places *bla*_NDM_ downstream of *ISAba125* using a rolling circle transposition mechanism, thereby providing potential evolutionary clues (Ilyina, 2012; Partridge and Iredell, 2012; Poirel et al., 2012).

Although antibiotic resistance via *bla*_NDM_ poses a significant potential threat to patients with bacterial infections, most research into the prevalence of *bla*_NDM_ in China has focused on individual strains, isolated events, or plasmid structures (Yang et al., 2017; Yang et al., 2018; Li et al., 2020; Li et al., 2020; Wang et al., 2020; Hu et al., 2022). Due to the lack of large-2scale data, the evolution, dissemination, and genetic dynamics of *bla*_NDM_ in China remain unknown. Furthermore, the overuse of antibiotics in China emphasizes the necessity for relevant research of the unique characteristics and patterns of the evolution of *bla*_NDM_ (Chang et al., 2019; Chen et al., 2019; Walley et al., 2021).

In this paper, the sequences of 1021 *bla*_NDM_-positive isolates were compiled, which included 954 bacterial genomes from NCBI and 67 newly collected bacterial genomes by our laboratory, effectively expanding early datasets of *bla*_NDM_-positive bacteria in NCBI and nearly doubling the number of isolates collected between 2010 and 2014.

Based on the collection, we conducted a comprehensive epidemiological analysis from multiple perspectives. Our key findings reveal that: (i) *Bla*_NDM_-positive bacteria exhibit a distinct livestock-environment-human transmission cycle, with significant clustered dissemination observed in economically developed and densely populated regions; (ii) The escalating misuse of antibiotics has led to a progressive increase in resistant strains, particularly those co-harboring *bla*_NDM_ and *tet(X)* genes, which demonstrate both expanding host ranges and enhanced resistance profiles; and (iii) Although bacteria co-harboring *bla*_NDM_ and *tet(X)* has currently only been detected in livestock and environmental samples, the established transmission patterns above strongly suggest imminent risks of human infection and potential community spread. Finally, referring to a previous study (Acman et al., 2022), we designed a more comprehensive analysis to intuitively show the differences and similarity of the *bla*_NDM_ contexts in various bacterial isolates and investigate the diversity of potential *bla*_NDM_-carrier transposon structures, which could assist the transfer of *bla*_NDM_ genes.

## Results

### Dataset of *bla*_NDM_-positive bacteria in China

A genomic dataset of 1021 *bla*_NDM_-positive isolates collected in China was assembled. On the one hand, 954 bacterial genomes are retrieved from the NCBI GenBank database (Benson et al., 2018) and the Reference Sequence database (O’Leary et al., 2016). The collection process is shown in the Methods section and their information is summarized in **Supplementary File 1**: *SF1_NCBI_954.xlsx*. Although sampling bias may be inherent, public genomic databases can provide the most comprehensive sequences (Wu et al., 2019). On the other hand, 67 bacterial genomes were collected by our laboratory, including 49 *bla*_NDM_-positive bacteria screened among 1403 carbapenem-resistant strains from 10 cities and 25 hospitals and 18 *bla*_NDM_-positive bacteria screened among 156 strains isolated from 16 hospital sewage samples. Genomic sequencing and assembly techniques are also shown in the Method section and their basic information is provided in **Supplementary File 2**: *SF2_newly_collected_67.xlsx*. The sequences of the 67 *bla*_NDM_-positive isolates were uploaded to the National Genomic Data Center.

**Figure 1:**
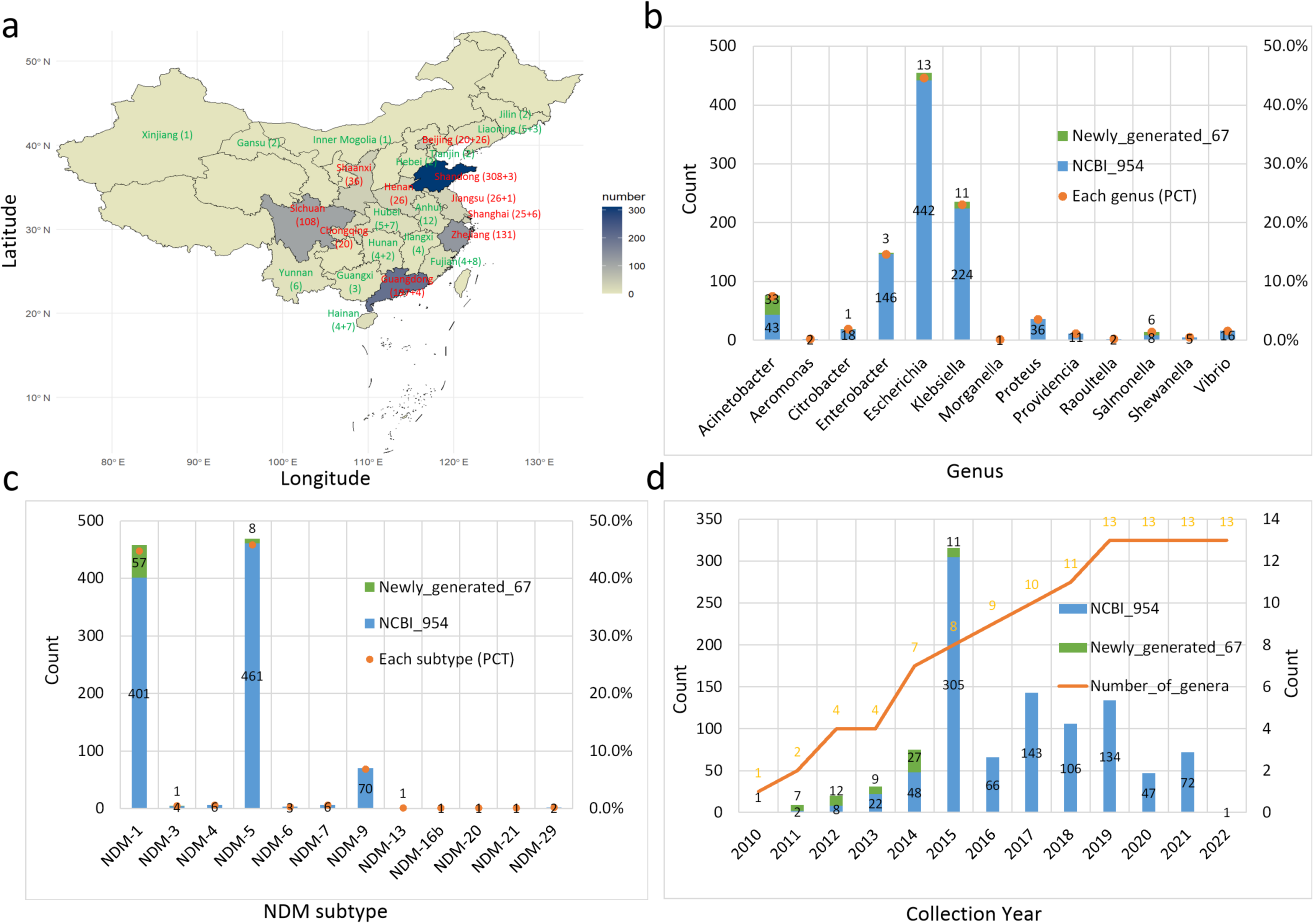
Composition of the China dataset of 1021 *bla*_NDM_-positive isolates. **(a)** Geographic distribution of *bla*_NDM_-positive assemblies. Provinces are colored by geographic region and the color reflects the number of isolates. The map was generated from coordinates using R software and the main provinces are highlighted. **(b)** Distribution of *bla*_NDM_-positive isolates at the genus level. **(c)** Distribution of *bla*_NDM_ subtypes. **(d)** Distribution of isolate collection years and the accumulative number of genera carrying the *bla*_NDM_ gene.

The dataset of the 1021 *bla*_NDM_-positive isolates includes the province, year of isolation, strain, genus, and *bla*_NDM_ subtype (**Figure 1**). The geographic distribution (provinces) of the isolates was collected and these with > 20 isolates are shown in red font and all others in green font (**Figure 1a**). The first number in the brackets denotes the dataset scale from the NCBI database, while the second number indicates the dataset scale of our collected bacterial genomes. The dataset is mainly distributed in the southwest, coastal, and central provinces of China. Among them, Shandong, Guangdong, Zhejiang, and Sichuan totally account for ~75%. The distribution of the bacterial isolates is categorized according to the taxonomy level of genera (**Figure 1b**). *Escherichia*, *Klebsiella*, and *Enterobacter* species account for >75% (PCT: percentage) of the dataset. Notably, the main genera worldwide are separately *Klebsiella*, *Escherichia*, and *Acinetobacter* (Acman et al., 2022), which slightly differs from the scenario in China. The main *bla*_NDM_ subtypes are separately *bla*_NDM-1_, *bla*_NDM-5_, and *bla*_NDM-9_ and the other nine subtypes only accounted for ~2% of the dataset (**Figure 1c**). Finally, we show the distribution of the collection year for the *bla*_NDM_-positive bacteria (**Figure 1d**). The number of the collected isolates gradually increased from 2010 to 2015, and then decreased and fluctuated after 2015, which is similar to the global tendency, where the global peak occurred in 2016 (Acman et al., 2022). At the same time, the accumulative number of genera that carry *bla*_NDM_ genes continuously increased from 2010 to 2019.

### Distributions of *bla*_NDM_-positive bacteria in China

**Figure 2:**
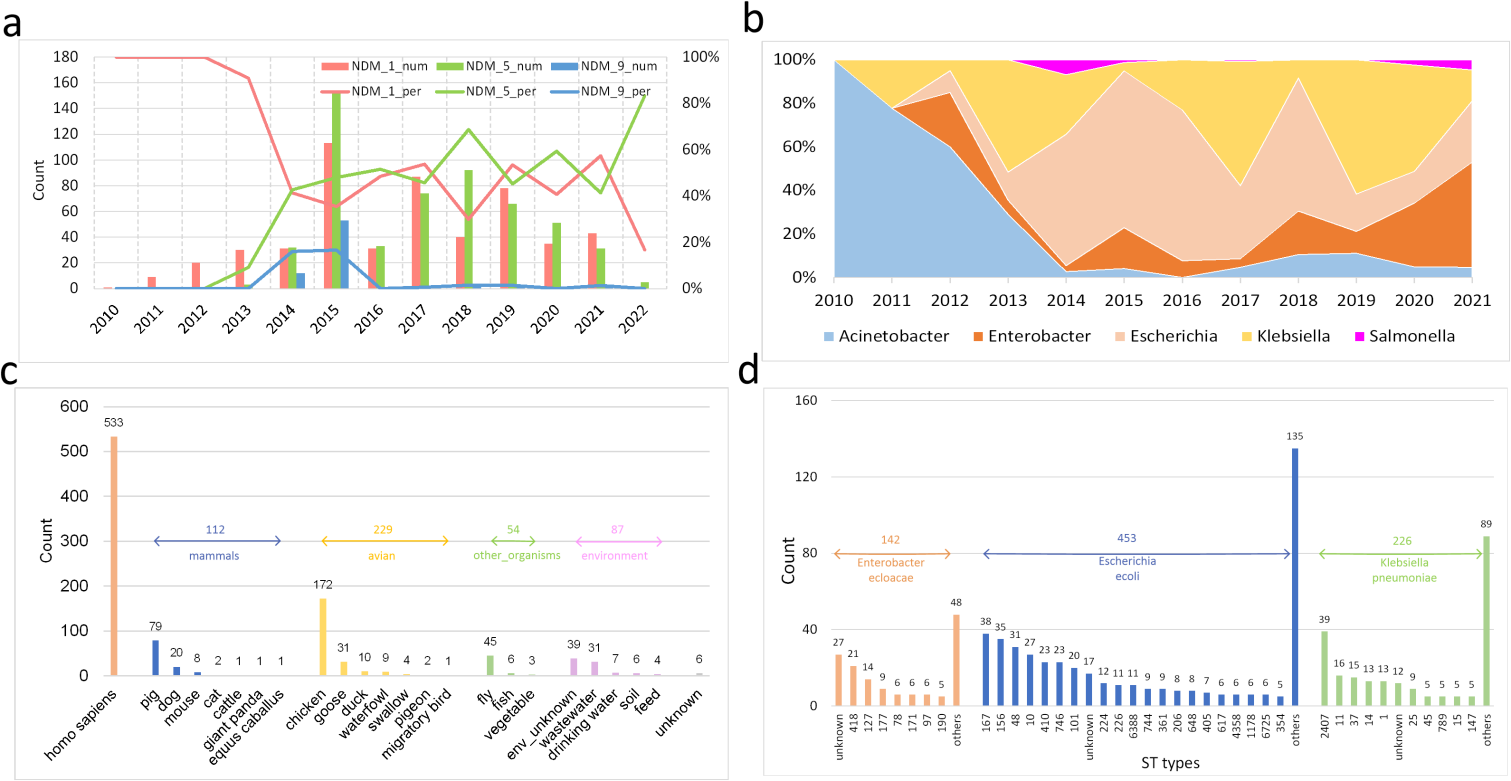
Distributions of the 1021 blaNDM-positive isolates in the China dataset **(a)** Temporal distribution of the three main bla_NDM_ subtypes. For each year, the number and percentage of each bla_NDM_ subtype are included. **(b)** Temporal distribution of the percentages of the five main bacterial genera carrying bla_NDM_-positive assemblies. **(c)** Collection sources distribution, which are categorized into five main groups with unknown source excluded. **(d)** ST types distribution of three main bacterial species by MLST.

From the perspective of temporal distribution, we firstly focus on the main subtypes of *bla*_NDM_, which are separately *bla*_NDM-1_, *bla*_NDM-5_ and *bla*_NDM-9_. *Bla*_NDM-1_ was the only subtype identified in China from 2010 to 2012 (**Figure 2a**). However, the *bla*_NDM-5_ and *bla*_NDM-9_ subtypes surfaced after 2013, with *bla*_NDM-5_ gradually surpassing *bla*_NDM-1_ as the most predominant subtype. Then, we focus on the main *bla*_NDM_-positive bacteria genera, which are separately *Acinetobacter*, *Enterobacter*, *Escherichia*, *Klebsiella*, and *Salmonella*. *Bla*_NDM_ initially occurred in *Acinetobacter*, which belongs to the *Moraxellaceae* family, before spreading to other genera, such as *Enterobacter*, *Escherichia*, and *Klebsiella*, which belong to the *Enterobacteriaceae* family (**Figure 2b**). After 2012, the ratios of *Enterobacter*, *Escherichia*, and *Klebsiella* gradually surpass the ratio of *Acinetobacter* among the *bla*_NDM_-positive genera. At the same time, the ratios of all *Enterobacter*, *Escherichia*, and *Klebsiella* isolates in NCBI (including both *bla*_NDM_-positive and *bla*_NDM_-negative isolates) are not increased compared to all *Acinetobacter* isolates (**Supplementary Figure 1**).

From the perspective of geographic distribution, as shown in **Supplementary Figure 2**, *bla*_NDM-1_ was identified as the dominant subtype in six provinces, i.e., Beijing, Shanghai, Zhejiang, Henan, Shaanxi, and Chongqing provinces, whereas *bla*_NDM-5_ was the subtype dominant in the other four provinces, i.e., Shandong, Jiangsu, Guangdong, and Sichuan provinces. The predominant genera carrying *bla*_NDM_ genes included *Escherichia* in Shandong, Jiangsu, and Guangdong provinces and *Klebsiella* in Beijing, Sichuan, and Shanghai provinces. *Enterobacter* emerged as the top genus in the central provinces of Shaanxi and Chongqing. Meanwhile, *Enterobacter* and *Escherichia* were the main carriers in Zhejiang province.

The temporal and geographic distributions of the other 9 *bla*_NDM_ subtypes and 10 genera are recorded in **Supplementary File 3**: SF3_NDM_subtype_genus_year_province_distribution.xlsx.

Moreover, sample origins are crucial for epidemiological analysis of bla_NDM_-positive bacteria. We categorized the samples into five groups: Homo sapiens, other mammals, avian species, other organisms, and environmental sources (**Figure 2c**). The majority of specimens were derived from Homo sapiens (i.e., patients; 52.2%), with the remaining predominantly originating from avian species (22.4%), other mammals (11.0%), and environmental reservoirs (8.5%). This distribution strongly suggests significant transmission of bla_NDM_-positive bacteria along the livestock-environment-human pathway. Notably, flies (4.4%) appear to serve as important vectors in this transmission network.

Finally, clonal analysis of the predominant bla_NDM_-positive bacterial species is crucial for understanding the epidemiological patterns. We conducted multilocus sequence typing (MLST) analysis of the three major species: *E. cloacae* (n=142), *E. coli* (n=453), and *K. pneumoniae* (n=226) (**Figure 2d**). Among the characterized sequence types, both *E. cloacae* and *E. coli* subtypes exhibited gradually decreasing prevalence without dominant clones, whereas *K. pneumoniae* showed a predominant ST2407 clone. Although this study primarily focused on transmission tracing at the species level, more detailed investigations of clonal-specific evolutionary dynamics and geospatial transmission patterns within each pathogen will be essential for future epidemiological analysis and disease prevention strategies.

### Geographic dissemination

Systematic phylogenetic analysis was conducted to investigate the dissemination of the main *bla*_NDM_-positive bacterial species throughout China. Four species with more than 15 isolates (i.e., *Proteus mirabilis*, *K. pneumoniae*, *Escherichia coli*, and *Enterobacter hormaechei*) were selected. The analysis process is described in the Methods section and the relevant configuration files are provided in availability of code (fasta_beast_setting).

**Figure 3:**
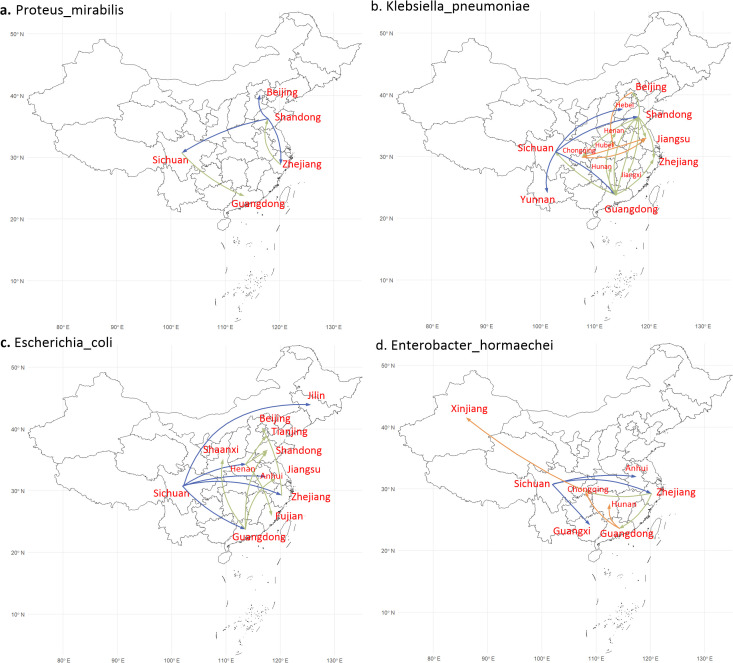
Geographic transfer of four bacterial species. The steps are indicated by different-colored arrows (blue, green and orange). **(a)** Geographic transfer of *P*. *mirabilis* (13 strains), which was first distributed from Shandong to Beijing, Zhejiang and Sichuan, and then diverted to Guangdong (from Sichuan) and back to Shandong (from Zhejiang). **(b)** Geographic transfer of *K. pneumoniae* (37 strains), which distributed from Sichuan to Shandong and Guangdong. **(c)** Geographic transfer of *E coli* (38 strains), which was distributed from Sichuan to Henan, Guangdong, and Zhejiang. **(d)** Geographic transfer of *E hormaechei* (24 strains), which was distributed from Sichuan to Zhejiang, Guangdong, and Chongqing.

To illustrate the dissemination process more clearly, different colors are utilized to separately mark the propagation paths (**Figure 3**). *P. mirabilis* was firstly discovered in Shandong and then spread to Beijing, Zhejiang, Sichuan, and Guangdong (**Figure 3a**). Meanwhile, both *K. pneumoniae* and *E. coli* were firstly detected in Sichuan and then spread to eastern China (**Figures 3b, c**). Similarly, *E*. *hormaechei* was also firstly discovered in Sichuan and then disseminated to southeast China and Xinjiang province (**Figure 3d**).

### *bla*_NDM_ and *tet(X)* genes

**Figure 4:**
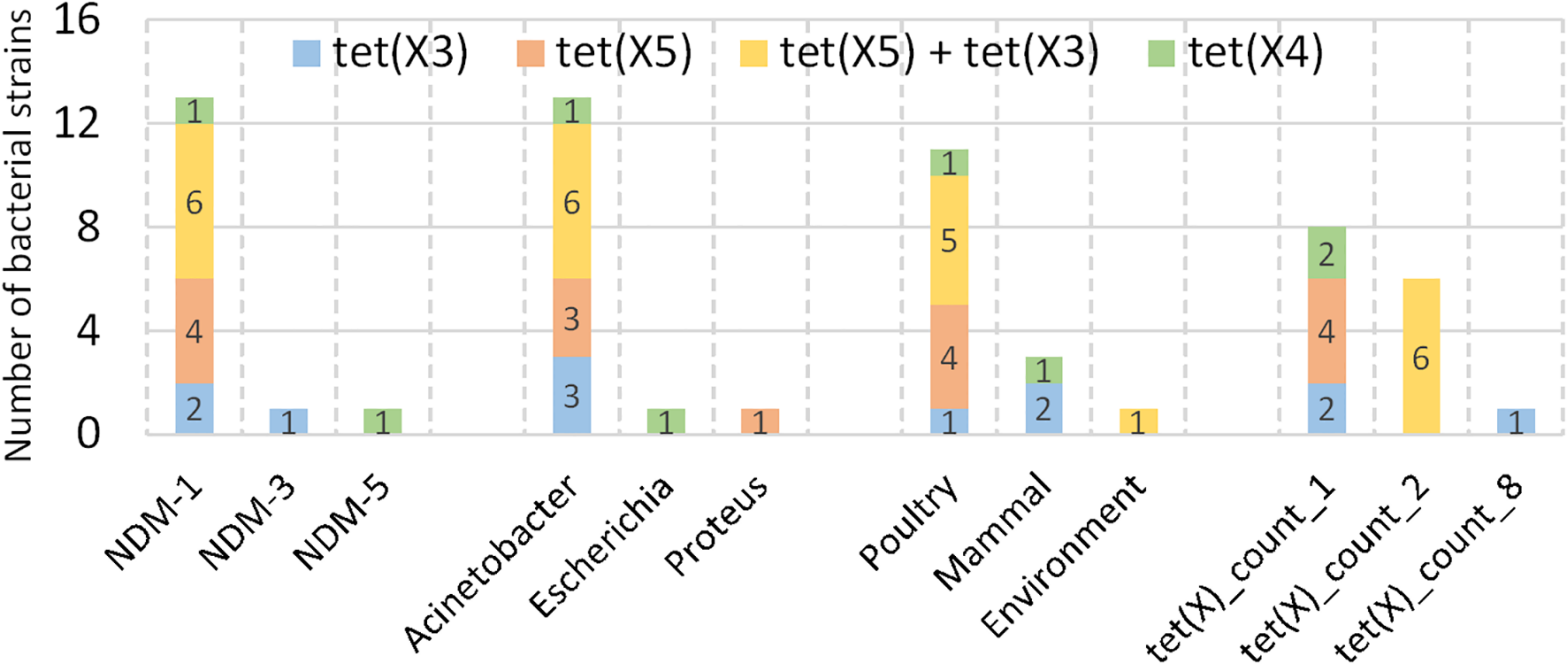
Co-occurrence of blaNDM and tet(X) genes. 15 strains co-harboring both *bla*_NDM_ and *tet(X)* genes are identified. For each subtype of *tet(X)*, various *bla*_NDM_ subtypes, genera, sample sources and the count of *tet(X)* emergency in a strain were collected.

The emergence of the plasmid-mediated high-level tigecycline resistance gene *tet(X)* has compromised the role of tigecycline as a “last-resort” antibiotic for treating carbapenem-resistant Gram-negative bacterial infections. Compared to the prototype *tet(X)*, the enzyme activity of *tet(X3)* and *tet(X4)* variants is significantly enhanced. It is observed that *tet(X3)/(X4)* genes are often associated with *bla*_NDM-1_ in various *Enterobacteriaceae* spp. and *Acinetobacter* and confer just a slight fitness cost on the isolates (He et al., 2020). Other groups made similar observations with these pathogens with diverse *tet(X)* genes mainly but not exclusively in the veterinary sphere (Hirabayashi et al., 2021; Chen et al., 2024; Yao et al., 2025). For in-depth investigation, we identified 15 resistant isolates co-harboring both *bla*_NDM_ and *tet(X)* genes from our collection of 1,021 strains (**Figure 4**). The *tet(X)* variants present in these samples were primarily three subtypes: *tet(X3)* and *tet(X4)* (MIC = 8 mg/L), along with *tet(X5)* (MIC = 4 mg/L) (Cui et al., 2021).

Notably, the strain GCA_015217905.1 harbored 8 *tet(X3)* genes and an unknown *tet(X)* variant, with all genes located in close genomic proximity. Additionally, six isolates carried two *tet(X)* variants, exclusively exhibiting the combination of *tet(X3)* and *tet(X5)*. Regarding *bla*_NDM_ variants, *tet(X)* genes predominantly co-occurred with *bla*_NDM-1_. There is only one isolate demonstrated coexistence of *bla*_NDM-3_ and *tet(X3)*, and another *bla*_NDM-5_ and *tet(X4)*. At the genus level, *Acinetobacter* represented the majority of isolates, while *Escherichia* and *Proteus* each accounted for only one isolate. Of particular note, the *bla*_NDM-5_ and *tet(X4)* co-occurrence was identified in the *Escherichia* isolate. These observations suggest potential genus-specific preferences for particular *bla*_NDM_ and *tet(X)* variant combinations. However, further validation of these characteristics will require additional sample collection and comprehensive analytical verification in subsequent studies. Regarding host origins, poultry constituted the primary reservoir (n=11), with minimal environmental contamination detected (n=1).

### Diversity of *bla*_NDM_ context genes

The *bla*_NDM_ context genes were extracted from the gbff files and automatically converted according to the Tn125 structure. More information of the *bla*_NDM_ context genes is provided in **Supplementary File 4**: SF4_954_67_NDM_downstream_7.xlsx.

**Figure 5:**
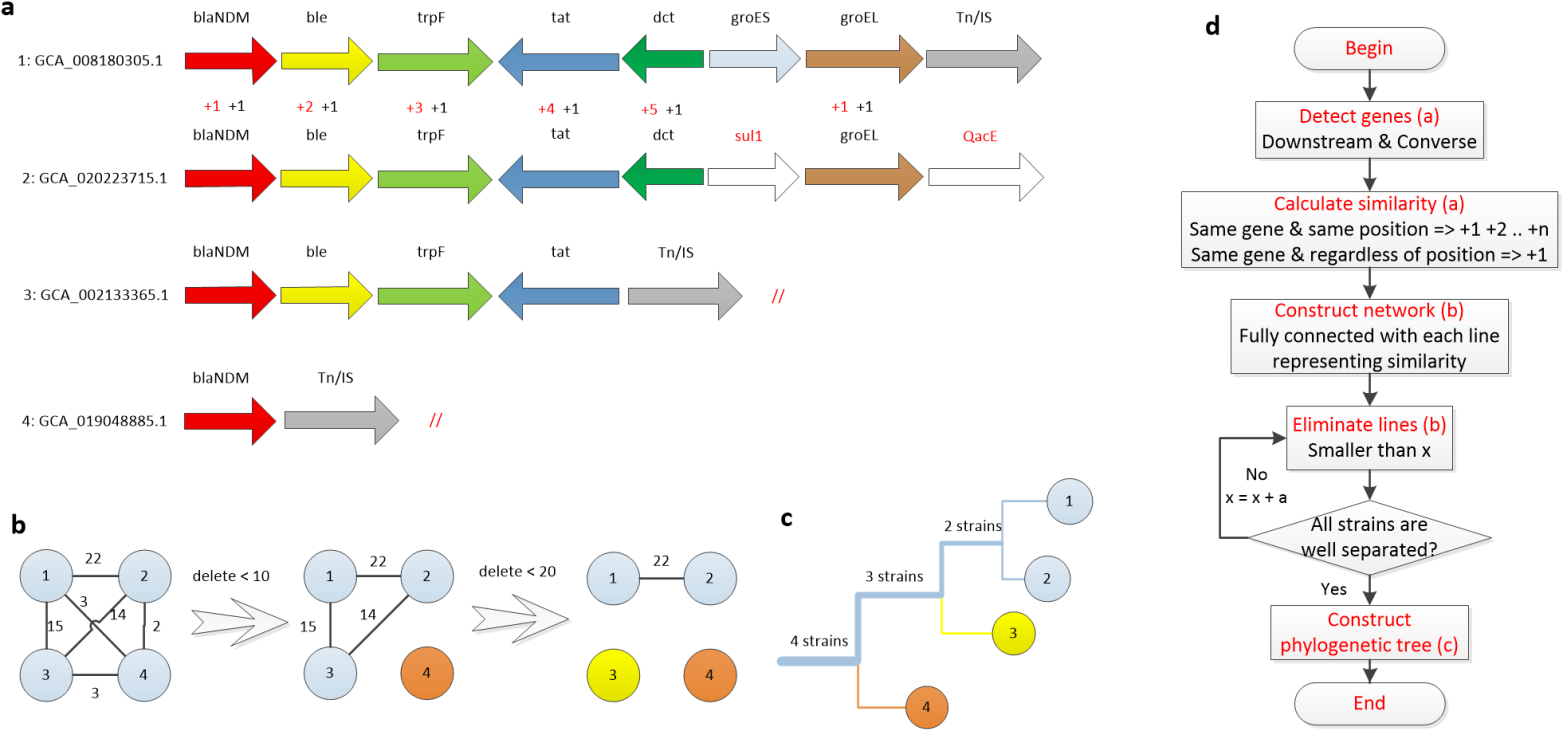
Similarity-based clustering of genes downstream of *bla*_NDM_ **(a)** Genes downstream of *bla*_NDM_ in four strains. **(b)** Fully connected network with each line representing the similarity of the connected two strains. Lines were gradually eliminated by a certain limitation until all strains were separated. **(c)** Clustering tree established according to results of step b where the thickness of each branch represents the number of strains. **(d)** The strain clustering method.

We utilize a clustering method similar to a previous study (Acman et al., 2022) to classify the *bla*_NDM_ context genes based on their structural similarities (**Figure 5**). It firstly measures the structural similarity between two *bla*_NDM_ downstream structures based on genes and positions (**Figure 5a**). Then, it generates a fully connected network where the node represents the bacterial isolate and the link represents similarity between two isolates (**Figure 5b**). By gradually eliminating links with increasing limitation, a clustering tree is established (**Figure 5c**). Specific process (**Figure 5d**) is described in the Methods section.

**Figure 6:**
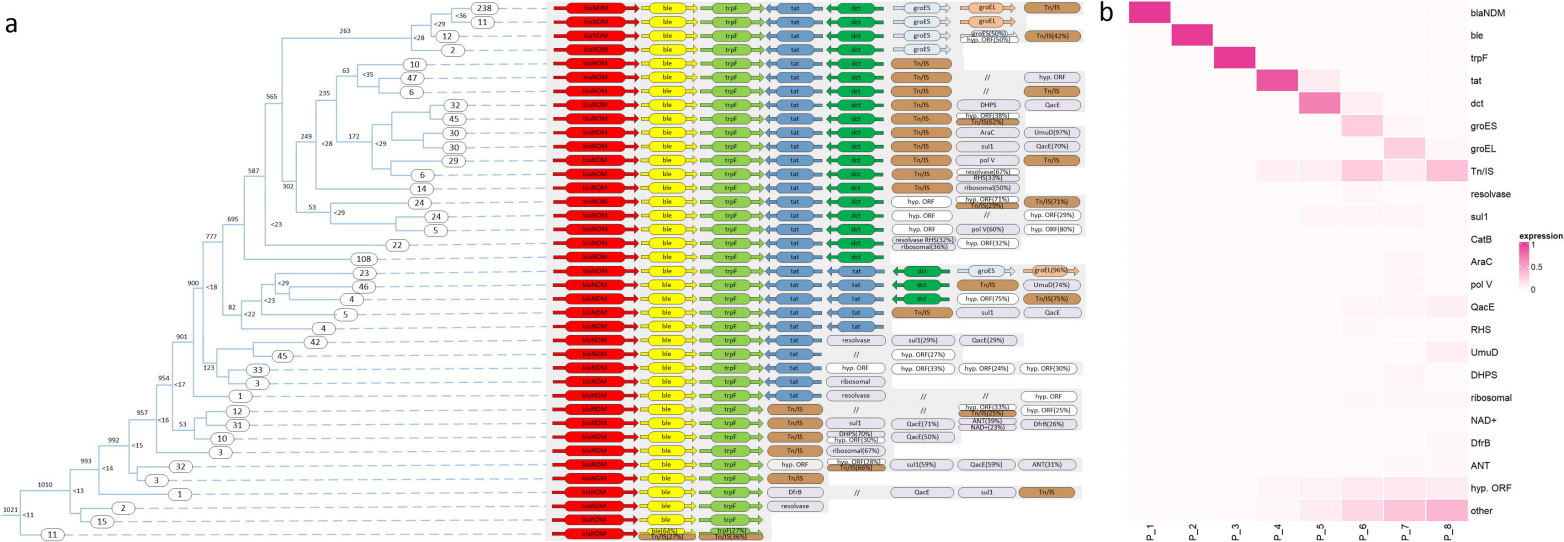
Details of *bla*_NDM_ context genes. **(a)** Clustering results of genes downstream of *bla*_NDM_. A tree was constructed in accordance with the method described in (**Figure 5**). The same gene in Tn125 context is marked with the same color. **(b)** A heat map of the relationships among different genes and the downstream positions from *bla*_NDM_.

The clustering results revealed that most of the *bla*_NDM_ context genes of the 1021 isolates were similar to the structure of Tn125 (**Figure 6a**). Among the 1021 isolates, 1010 (99%) carried a combination of the *bla*_NDM_, *ble*, and *trpF* genes; 88% (901) had a more extended group of genes, which included *bla*_NDM_, *ble*, *trpF*, and *tat*; 695 (68%) contained a gene structure consisting of *bla*_NDM_, *ble*, *trpF*, *tat*, and *dct* genes; 249 (24%) had a complete gene structure equivalent to Tn125, which excludes Tn/IS (Transposase/Insertion Sequence); 82 (8%) carried duplicated *tat* genes, while 95% of these also carried the functionally related genes *groES* and *groEL*. Other genes frequently found downstream of *bla*_NDM_ included *Resolvase*, *catB*, *dfrB*, *qacE*, *sul1*, *ANT*, *DHPS*, *ribosomal*, *NAD+*, *umuD*, *polV*, *RHS*, and *AraC*, which were identified in >2% of the isolates.

A heatmap was constructed to visualize the diversity of genes located at different positions downstream of *bla*_NDM_ (**Figure 6b**). Position P_4 was the primary point of gene diversity. Notably, from P_4 to P_8, the likelihood of gene diversity increased. The frequency of Tn/IS also increased from P_4 to P_8, with peak value occurring at P_6 (the position of *groES* in *Tn125*) and P_8 (the position of Tn/IS in Tn125).

### Potential *bla*_NDM_-carrier transposons

To find the potential *bla*_NDM_-carrier transposons, further in-depth analysis of the characteristics of the Tn/IS elements upstream and downstream of *bla*_NDM_ was conducted. Two upstream and nine downstream genes of *bla*_NDM_ were extracted from the gbff files (**Supplementary File 5**). Especially, the positions of the Tn/IS elements were categorized based on the family and similarity in **Supplementary File 6**: SF6_IS_like.xlsx.

Totally, seven primary Tn/IS elements were identified, which included *ISAba125*, *IS5*, *IS91*, *IS6*, *Tn3*, *ISL3*, and *IS1* and all infrequent Tn/IS elements were categorized into a single group called “others” (**Supplementary Figure 3**). Besides, the distribution of the seven main Tn/IS among different provinces, genera and collection years are also analyzed. Additional information is provided in **Supplementary File 7**: SF7_954_67_IS_distance_genus_province_year.xlsx.

**Figure 7:**
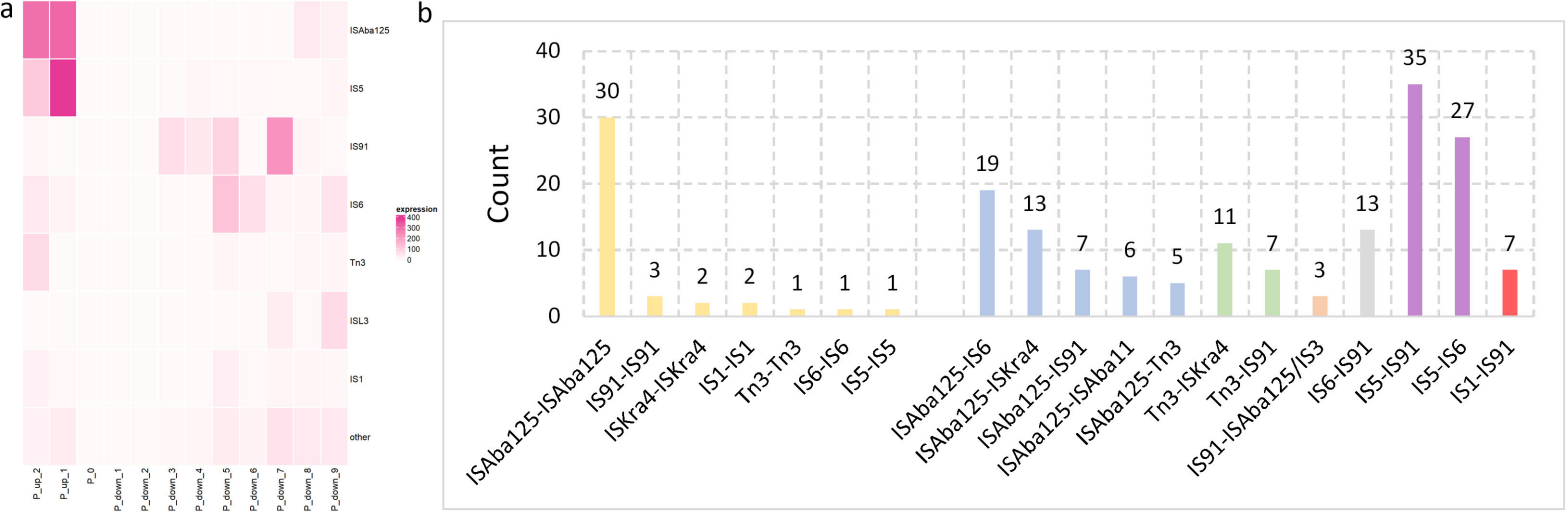
Positions and relationships of the Tn and IS elements downstream and upstream from blaNDM. **(a)** A heat map of the relationship among the 7 main Tn/IS types as well as other types, and corresponding positions from *bla*_NDM_. P_up_2 represents the second upstream gene from *bla*_NDM_, P_0 represents the position of *bla*_NDM_, and P_down_1 represents the first downstream gene from *bla*_NDM_. **(b)** Tn/IS pairs containing the *bla*_NDM_, *ble*, *trpF*, *tat*, *dct groES* and *groEL* genes were separated into two main groups: one including the same Tn or IS in the Tn/IS pair (yellow bars) and a second including different Tn or IS in the Tn/IS pair (blue, green, orange, grey, and purple bars with each color representing one upstream Tn/IS type).

A heat map was generated to clearly identify the possible positions of the Tn/IS elements upstream and downstream of *bla*_NDM_ (**Figure 7a**). *ISAba125* and *IS5* were primarily situated upstream of *bla*_NDM_ (up_1 and up_2), while *IS6* and *Tn3* occurred less frequently (*IS6*: up_1 and up_2; *Tn3*: up_2). Other Tn/IS genes rarely occurred upstream of *bla*_NDM_. In contrast, the downstream region of *bla*_NDM_ was more complex. Specifically, *IS91* was the most common gene and primarily located at four positions (down_3, down_4, down_5, and especially down_7). Meanwhile, *IS6* frequently appeared downstream at positions down_5, down_6, and down_9. *ISAba125* and *ISL3* were less prevalent than *IS91* and *IS6* but more frequent than *IS1* and *IS5* downstream of *bla*_NDM_. These results demonstrate that the Tn/IS elements were asymmetrically distributed both upstream and downstream of *bla*_NDM_.

Further comprehensive investigation into potential *bla*_NDM_-carrier transposons was conducted based on the distribution of the Tn/IS elements. The complete structure of Tn125 comprises seven genes between two *ISAba125* elements: *bla*_NDM_, *ble*, *trpF*, *tat*, *dct*, *groES*, and *groEL*. There were 7 symmetrical combinations of Tn/IS pairs (yellow bars), which included *ISAba125*-*ISAba125*, *IS91*-*IS91*, *ISKra4*-*ISKra4*, *IS1*-*IS1*, *Tn3*-*Tn3*, *IS6*-*IS6*, and *IS5*-*IS5* (**Figure 7b**). Notably, the *ISAba125*-*ISAba125* combination (Tn125) was the most common and *Tn3*-*Tn3* (*IS3000*-*IS3000*) and *IS6*-*IS6* (*IS26*-*IS26*) were previously reported as novel *bla*_NDM_-harboring transposons (Campos et al., 2015; Zheng et al., 2021). There were also many other asymmetrical Tn/IS pairs with relatively high frequencies, especially the *IS5*-*IS91* pair, which was observed 35 times, even higher than the frequency of *ISAba125*-*ISAba125*. More information is provided in **Supplementary File 8**: SF8_954_67_transposon_complete.xlsx.

## Discussion

*Bla*_NDM_-positive bacteria are predominantly found in Asia, particularly mainland China (Acman et al., 2022). In this study, the basic characteristics, evolution, geographic dissemination, and genetic dynamics of *bla*_NDM_-positive bacteria in China were analyzed to help develop potential strategies to control the proliferation of *bla*_NDM_ and prevent the development of drug-resistant bacteria. Although the analysis results could bias due to selection for sequencing, we strive to collect a sufficiently diverse range of data to ensure the validity of the results. Totally, 954 bacterial strains were retrieved from the NCBI database and 67 new *bla*_NDM_-positive bacteria genomes were identified by our laboratory, increasing the dataset by 7%. Especially, the 67 genomes effectively enhanced the NCBI dataset of early years (from 2010 to 2014) and early carrier of the *bla*_NDM_ gene (*Acinetobacter*), offering insights into the complete evolution and dissemination process of *bla*_NDM_-positive bacteria in China.

From the perspective of geographic analysis, the majority of *bla*_NDM_-positive isolates were concentrated in economically developed and densely populated coastal, middle, and southwest provinces of China, including Guangdong, Zhejiang, Shanghai, Jiangsu, Shandong, Beijing, Sichuan, Chongqing, Shaanxi, and Henan (**Figure 1a**). The geographic dissemination analysis shows that the *bla*_NDM_-positive bacterial strains might be frequently transmitted from Sichuan to the coastal provinces (**Figure 3**). Although potential bias cannot be ruled out, the dissemination results reflect the trend of *bla*_NDM_ spread to some extent. Compared with Beijing, Shanghai, and Guangdong, Sichuan is also a province with high population density. At the same time, it is near to South Asia, where the first *bla*_NDM_-positive bacterial isolate was discovered, which makes the dissemination results more reliable (Yong et al., 2009). The results of the geographic analysis indicate that *bla*_NDM_-positive bacteria are mainly distributed in economically developed and densely populated provinces in China, which will severely constrain people’s health and urban economic development. Therefore, we need to pay special attention to the prevention and control measures in these cities, as well as focus on preventing and controlling the main transmission route from west to east in China.

From the perspective of temporal analysis, there was a dramatic increase in *bla*_NDM_-positive bacteria from 2010 (first emergence in China) and the peak in 2015, which was followed by a steep decrease (**Figure 1d**). The consumption data of antibacterial drugs from 2012 to 2020 were obtained from the pharmaceutical database of China Pharmaceutical Industry Information Center and summarized in **Supplementary Figure 4**. The usage of carbapenems increased sharply from 2012 to 2015/2016, eventually stabilizing at a high level, suggesting a link between the increased prevalence of *bla*_NDM_-positive bacteria and continued overuse of antibiotics. Additionally, from 2010 to 2019, there was an increase in the cumulative number of genera carrying *bla*_NDM_ genes, demonstrating that the *bla*_NDM_ gene has continued dissemination among an increasing number of bacterial genera (**Figure 1d**). Among these genera, *Escherichia*, *Klebsiella*, and *Enterobacter*, members of the *Enterobacteriaceae* family, were identified as the most prevalent *bla*_NDM_-positive genera in China (**Figure 1b**), highlighting the importance of *Enterobacteriaceae* for the *bla*_NDM_ transmission among humans (Perez and Bonomo, 2019). The temporal analysis shows that although the *bla*_NDM_ resistance gene was initially detected in an *Acinetobacter* isolate, a shift has been observed from Acinetobacter to genera *Escherichia*, *Klebsiella*, and *Enterobacter* within the family Enterobacteriaceae (**Figure 2b**). Finally, the dominant *bla*_NDM_ subtypes in China are separately *bla*_NDM-1_, *bla*_NDM-5_ and *bla*_NDM-9_ (**Figure 1c**). Nowadays, the prevalence of *bla*_NDM-1_ has decreased over time, gradually being replaced and possibly being surpassed by *bla*_NDM-5_ (**Figure 2a**). The results of the temporal analysis indicate that antibiotic abuse is one of the main causes of the growth of antibiotic-positive bacteria in China. Therefore, we need to avoid the overuse of antibiotics and focus on preventing the spread of *bla*_NDM_-positive genes especially caused by Enterobacteriaceae bacteria, as well as the two dominant subtypes genes *bla*_NDM-1_ and *bla*_NDM-5_.

The current study has limitations in spatiotemporal sampling heterogeneity, with significantly more samples collected in 2015 and from eastern coastal provinces compared to other time periods and regions (**Figures 1a, b**). Such sampling bias may potentially impact key conclusions in several aspects: (i) Distortion of transmission pathway inference - over-representation of developed areas could lead to overestimation of hospital/community-based transmission while underestimating cross-species transmission in agricultural regions; (ii) Miscalculation of evolutionary rates - disproportionate sampling during peak years (e.g., 2015) may introduce deviations in estimating genetic mutation accumulation rates; and (iii) Compromised strain representativeness - insufficient sampling in less-developed areas might miss locally prevalent clones and underestimate their importance in transmission networks. Nevertheless, several significant conclusions can still be drawn from the current dataset analysis. For example, epidemiological investigation of *bla*_NDM_-positive bacterial hosts (**Figure 2c**) revealed that the livestock-environment-human transmission chain likely serves as the intrinsic driver of the observed spatiotemporal dissemination patterns. However, to facilitate in-depth epidemiological analysis and effective infection control, future studies must prioritize detailed transmission tracing focusing on distinct clonal lineages (**Figure 2d**) of the predominant bacterial species.

Since *tet(X)* genes are well-established to confer a high-level resistance to tetracyclines (Sun et al., 2019) and were reported to be associated with low fitness cost, though certainly in a strain-specific fashion (He et al., 2019; Jiang et al., 2021; Xu et al., 2021; Tang et al., 2022), the carriage of the *tet(X)* genes should be beneficial to many multidrug resistance (MDR) pathogens in a high antibiotic environment. The screened samples spanned from 2015 to 2020 (**Figure 4**). Early isolates (2015–2018) exclusively belonged to the *Acinetobacter* genus and carried only *tet(X3)* and *tet(X4)* variants. However, in 2019 and 2020, we detected emergent *Proteus* and *Escherichia* genera harboring the novel *tet(X4)* variant. Notably, *tet(X4)* exhibits the highest enzymatic activity among all *tet(X)* subtypes, conferring the most significant resistance phenotype (Cui et al., 2021). Furthermore, its co-occurrence with *bla*_NDM-5_—a carbapenemase variant with enhanced resistance—amplifies clinical concerns. These findings collectively demonstrate: 1) Expanding host diversity: Gradual co-emergence of *bla*_NDM_ and *tet(X)* across new bacterial genera (*Proteus*, *Escherichia*), indicating their accelerated interspecies dissemination; 2) Directed molecular evolution: Resistance genes are evolving toward higher enzymatic activity and stronger resistance profiles (e.g., *tet(X4)* and *bla*_NDM-5_ synergy).

While no human-derived co-resistant isolates have been identified yet, our prior research confirmed the livestock–environment–human transmission route for *bla*_NDM_-positive bacteria. Given the confirmed presence of *bla*_NDM_/*tet(X)* co-carriage in livestock and environmental reservoirs, potential zoonotic transmission remains plausible. This impending threat could severely compromise future clinical treatment options, necessitating urgent implementation of: 1) Genomic surveillance programs to track resistance gene flow; 2) Preemptive containment measures targeting agricultural and environmental reservoirs; 3) Stewardship protocols for last-resort antibiotics (e.g., tigecycline/carbapenems).

From the perspective of molecular analysis, the genetic dynamics of the *bla*_NDM_ context genes were analyzed, with a particular focus on the IS/Tn elements that facilitate the transfer of *bla*_NDM_ among bacteria. The genetic structure of 24% had the complete Tn125 structure and of >99% featured a combination of *bla*_NDM_, *ble*, and *trpF*, most of which are interrupted by Tn/IS (**Figure 6a**). The heat map also reflects that the gene diversity mainly emerged from P_4, and from P_4 to P_8, the gene diversity increased. Seven major Tn/IS, i.e., *ISAba125*, *IS5*, *IS91*, *IS6*, *Tn3*, *ISL3*, and *IS1*, are typically found either upstream or downstream of *bla*_NDM_ (**Figure 7a**). Of these, Tn125, the combination of two *ISAba125* elements, is the primary transposon (**Figure 7b**). The other six potential transposons (yellow bars) have a low occurrence rate, and among them, the *Tn3*-*Tn3* and *IS6*-*IS6* pairs have been confirmed as transposons in earlier reports (Campos et al., 2015; Zheng et al., 2021). The formation of a transposon necessitates symmetry of Tn/IS elements at both ends. However, asymmetrical distribution of Tn/IS elements both upstream and downstream of *bla*_NDM_ is also very common, the emergency frequency of which are even higher than the reported transposons *Tn3*-*Tn3* and *IS6*-*IS6*. The results of molecular analysis indicate that there are potential various structures of *bla*_NDM_-carrier transposon, which could facilitate the transmission and dissemination of *bla*_NDM_ gene. At the same time, the upstream and downstream genes of *bla*_NDM_ are primarily structured around Tn125. However, the further away from the *bla*_NDM_ gene, the more diverse the genes tend to be, which is of high possibility due to the insertion of Tn/IS. Therefore, when preventing the spread of *bla*_NDM_ genes, it is necessary to consider both the diversity of multiple transposon structures and *bla*_NDM_ context genes.

## Methods

### Data collection

The NCBI database was comprehensively searched to identify all genera carrying *bla*_NDM_ and all genomic sequences of sufficient assembly level (≥scaffold) were downloaded. Within these genera, strains carrying *bla*_NDM_ were identified and further filtered based on the prevalence in China.

Point mutations can result in different, but similar, *bla*_NDM_ subtypes. Therefore, the *bla*_NDM-1_ subtype was selected as a reference to search the NCBI database with the criteria of coverage >10% and identity >80%. The search results identified 2,894 potentially relevant species distributed across 34 genera (*SF1: NCBI_collection*), primarily in the family *Enterobacteriaceae*, order *Enterobacterales*, class *Gammaproteobacteria*, and phylum *Proteobacteria*. As of November 10, 2022, the NCBI database included 57,017 genomes (worldwide) for these species with 10,404 specifics to China. Among the 10,404 genomes specific to China (accession numbers are provided in *SF1: 10404_China*), 954 that fulfilled the following three rigorous criteria were selected as the NCBI dataset used in this study to make the analysis results more credible and reliable.

• Assembly level higher than contig and quality (=completeness–5*contamination) should be greater than 95%.
• Accurate collection year and location (province) data.
• Inclusion of the complete *bla*_NDM_ sequence (813 bp).

Our collected bacterial genomes were extracted from cultured bacteria using the QIAamp DNA Mini Kit (Qiagen, Inc., Valencia, CA, USA) and sequenced by Novogene Co., Ltd. (Beijing, China) using the Illumina HiSeq 2500 platform (Illumina, Inc., San Diego, CA, USA) with an insert size of 350 bp. The genome was assembled *de novo* using the SOAPdenovo genome assembler (v2.04) (Li et al., 2010) with an average coverage of 110 fold. Scaffolding and gap filling were performed using SSPACE and GapFiller, respectively (Boetzer et al., 2011; Boetzer and Pirovano, 2012).

### Geographic dissemination

To ensure adequate data for geospatial analysis, four species with >15 collected isolates were selected from the 1021 *bla*_NDM_-positive isolates. Ultimately, 13, 37, 38, and 24 isolates of *P. mirabilis*, *K. pneumoniae*, *E. coli*, and *E. hormaechei*, respectively, collected in different years and from different regions (provinces) were used for geospatial analysis (more information is provided on https://github.com/wr-sky/NDM:fasta_beast_setting/List_of_four_species.txt). *BEAUti* software in *Beast* (Bouckaert et al., 2014) was utilized to extract the region and year of collection of each species. The parameters included the substitution model (Blosum62), clock type (uncorrelated relaxed clock, relaxed distribution: lognormal), and other default options. Complete information on the fasta files and BEAUti settings are provided on https://github.com/wr-sky/NDM:fasta_beast_setting. Multiple runs were conducted using Beast until each parameter exceeded 200 when combined in Tracer log files to assure the analysis results more reliable and trustworthy. Multiple tree files were combined using LogCombiner software (https://beast.community/logcombiner) and then simplified by TreeAnnotator software (https://beast.community/treeannotator). The geographic dissemination of each species was simulated and calculated by SpreaD3 (Spatial Phylogenetics Reconstruction of Evolutionary Dynamics using Data-Driven Documents (D3)) (Bielejec et al., 2016) with the geographic data of the province in China as input (https://github.com/wr-sky/NDM:fasta_beast_setting*/all_pro_pos.txt*&*china.json* ).

### Clustering of *bla*_NDM_ context genes

The complete process of the clustering method is shown in **Figure 5d**. Initially, a set of seven downstream genes were be automatically extracted from the gbff file and formatted by the program. Then, manual inspection was applied for quality control. The final sequencing results of the downstream genes are provided in SF4: SF4_954_67_NDM_downstream_7.xlsx. Next, the similarity of each pair of gene sequences was measured based on the following principles:

• For the same gene in the same position, add 1, 2, and n scores in sequence. Once two genes in the same position are different, add score from 1 again.
• For the same gene, the score was increased by 1, regardless of the position.

A representative calculation of the similarity of the sequences of GCA_008180305.1 and GCA_020223715.1 is shown (**Figure 5a**). First, the score of each corresponding gene at the same position was calculated (red font). Since the five genes (*bla*_NDM_, *ble*, *trpF*, *tat*, and *dct*) are continuously aligned, scores of 1, 2, 3, 4, and 5 were added, respectively. Because *groEL* is not continuous with the other five genes, a score of 1 was added from the beginning. Next, the number of the same gene in both sequences was calculated and the corresponding numbers were added (black font). Finally, the similarity between these two gene sequences was assigned a score of 22.

After determining the similarity of each pair of sequences, a fully connected network was established where each node represents a sequence and each link represents the respective similarity score (**Figure 5b**). Subsequently, links were gradually eliminated with increasing similarity score limitations, resulting in a clustering tree. The first branch of the tree structure was formed by deleting links smaller than 10, which separated node 4 (**Figure 5c**). In the second branch, links smaller than 20 were deleted, resulting in the isolation of node 3. The remaining nodes 1 and 2 form the leaves of the last branch. The method generated a clustering tree of 1021 *bla*_NDM_-positive isolates (**Figure 6a**). Tn125 is marked in the same color, Tn/IS in brown, hypothetical proteins (*hyp.ORF*) in white, and all other 13 frequently emerged genes in purple.

## Data Availability

The datasets presented in this study can be found in online repositories. The names of the repository/repositories and accession number(s) can be found below: https://ngdc.cncb.ac.cn/, National Genomic Data Center PRJCA017599.
