# Supplementary material for: Evolution, dissemination, and genetic dynamics of the carbapenem resistance gene bla NDM in China
Source: Front Cell Infect Microbiol. 2025 Aug 11;15:1608826. doi: 10.3389/fcimb.2025.1608826 (PMC12375619; doi:10.3389/fcimb.2025.1608826)
Supplement: Supplementary file 1 [file DataSheet1.pdf]

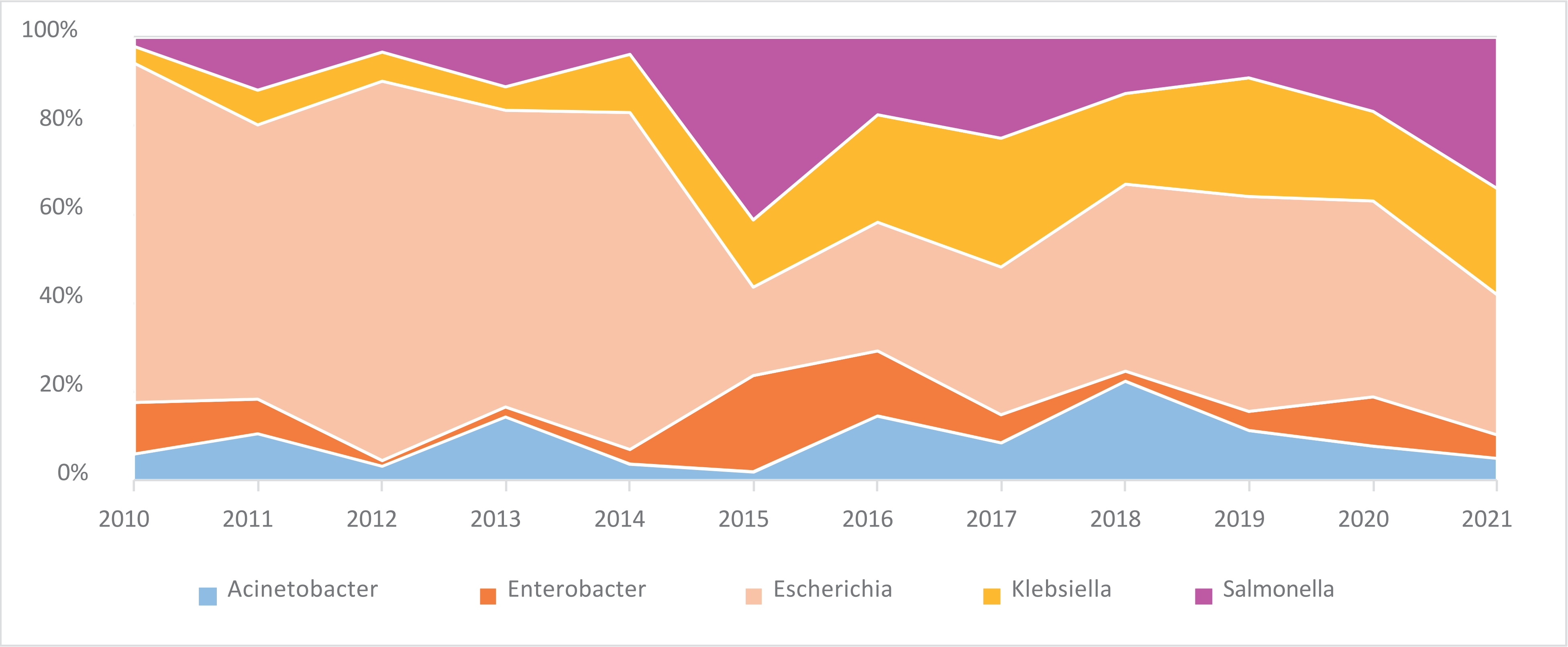


**Supplementary Figure 1:** Temporal distribution of the percentages of the five bacterial genera samples upload to NCBI. The samples include the strains with NDM and without NDM gene.

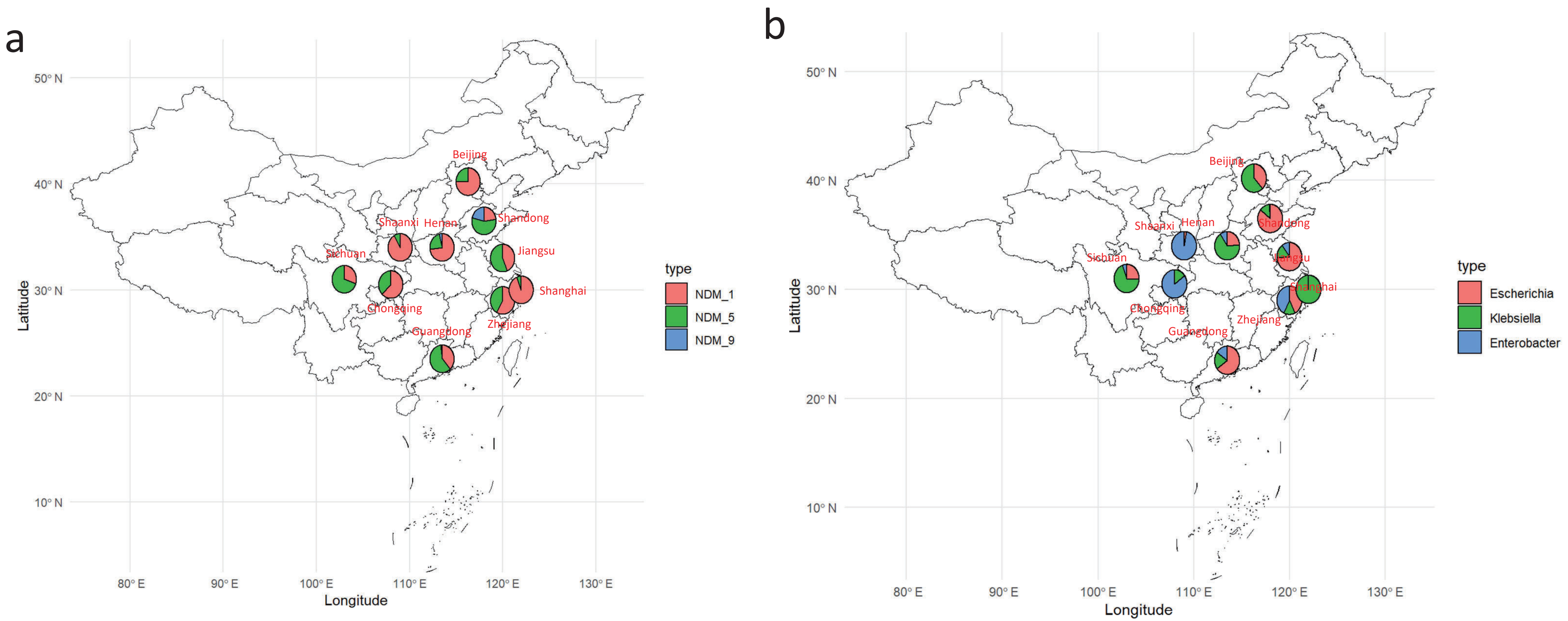


**Supplementary Figure 2: Geographic distribution of main NDM subtypes and NDM-positive bacterial genera.**

**a)** Geographic distribution of the three main NDM subtypes. A map of the coordinates was generated using R software. The 11 main provinces are indicated by different colors to show the ratios of different NDM subtypes.

**b)** Geographic distribution of the three main bacterial genera containing NDM-positive assemblies. A map of the coordinates was generated using R software. The 11 main provinces are indicated with different colors to show the ratios of different bacterial genera.

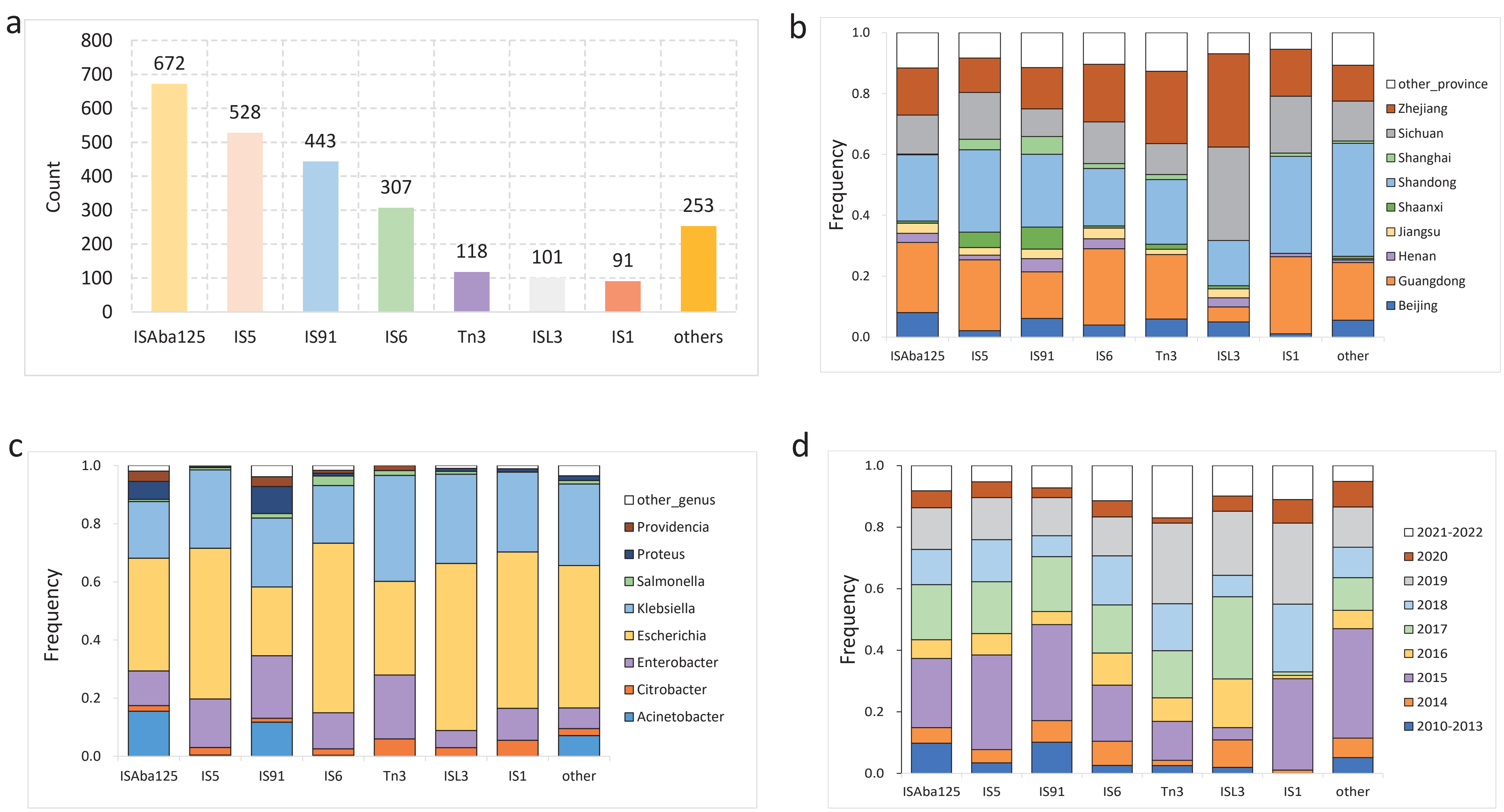


**Supplementary Figure 3: Statistical data of the Tn and IS elements upstream and downstream of NDM. a)** The number of isolates with the 7 main Tn/IS types as well as other types. The statistic scope included 2 and 9 genes upstream and downstream, from NDM, respectively. **b)** Geographic distribution of the 7 main Tn/IS types as well as other types. Other provinces included Anhui, Gansu, Guangxi, Hainan, Hebei, Hunan, Inner Mongolia, Jiangxi, Jilin, Liaoning, Tianjin, Xinjiang, Yunnan, Hubei, Fujian, and Chongqing. **c)** Distribution of the 7 main Tn/IS types as well as other types at the genus levels. Other genera included Raoultella, Shewanella, Vibrio, Morganella, and Aeromonas. **d)** Distribution of the year of collection of the 7 main Tn/IS types as well as other types.

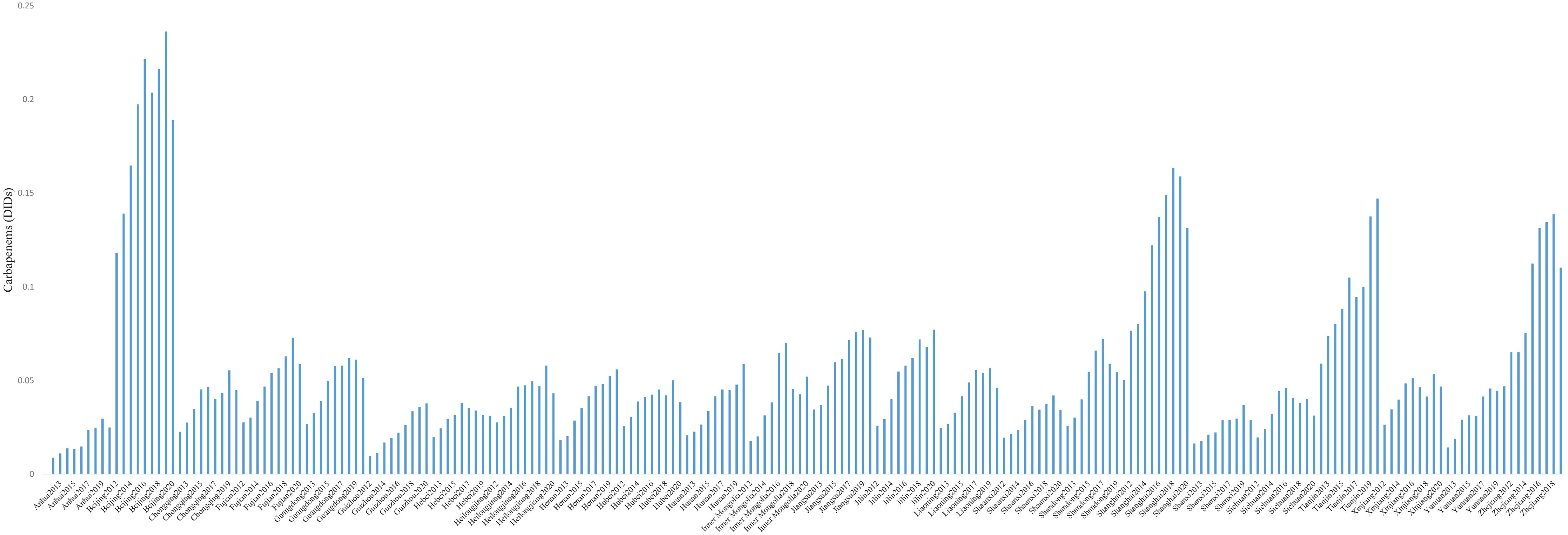


**Supplementary Figure 4:** The consumption data of antibacterial drugs from 2012 to 2020, which was obtained from the pharmaceutical database of China Pharmaceutical Industry Information Center.
