# Supplementary material for: Evolution, dissemination, and genetic dynamics of the carbapenem resistance gene bla NDM in China
Source: Front Cell Infect Microbiol. 2025 Aug 11;15:1608826. doi: 10.3389/fcimb.2025.1608826 (PMC12375619; doi:10.3389/fcimb.2025.1608826)
Supplement: Supplementary file 2 [file DataSheet2.pdf]

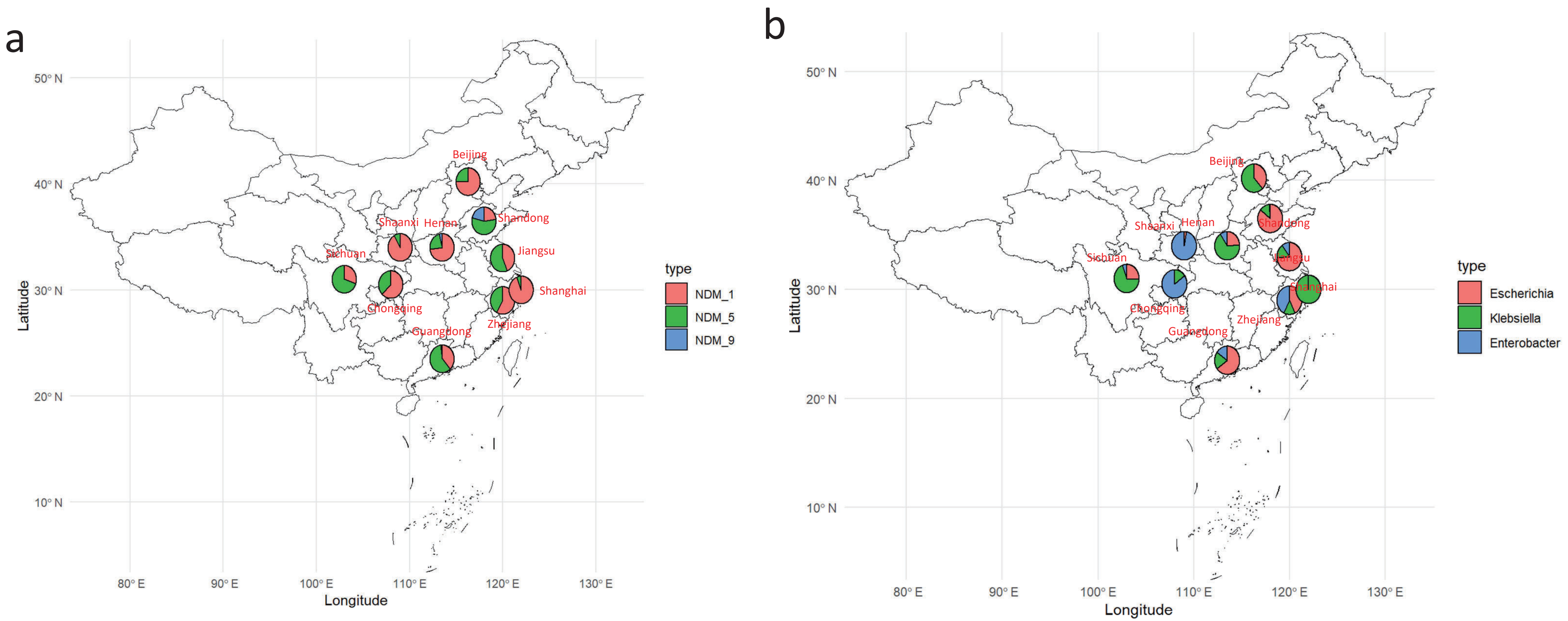

Supplementary Figure 2: Geographic distribution of main NDM subtypes and NDM-positive bacterial genera.

a) Geographic distribution of the three main NDM subtypes. A map of the coordinates was generated using R software. The 11 main provinces are indicated by different colors to show the ratios of different NDM subtypes.

b) Geographic distribution of the three main bacterial genera containing NDM-positive assemblies. A map of the coordinates was generated using R software. The 11 main provinces are indicated with different colors to show the ratios of different bacterial genera.
