# Supplementary material for: Evolution, dissemination, and genetic dynamics of the carbapenem resistance gene bla NDM in China
Source: Front Cell Infect Microbiol. 2025 Aug 11;15:1608826. doi: 10.3389/fcimb.2025.1608826 (PMC12375619; doi:10.3389/fcimb.2025.1608826)
Supplement: Supplementary file 3 [file DataSheet3.pdf]

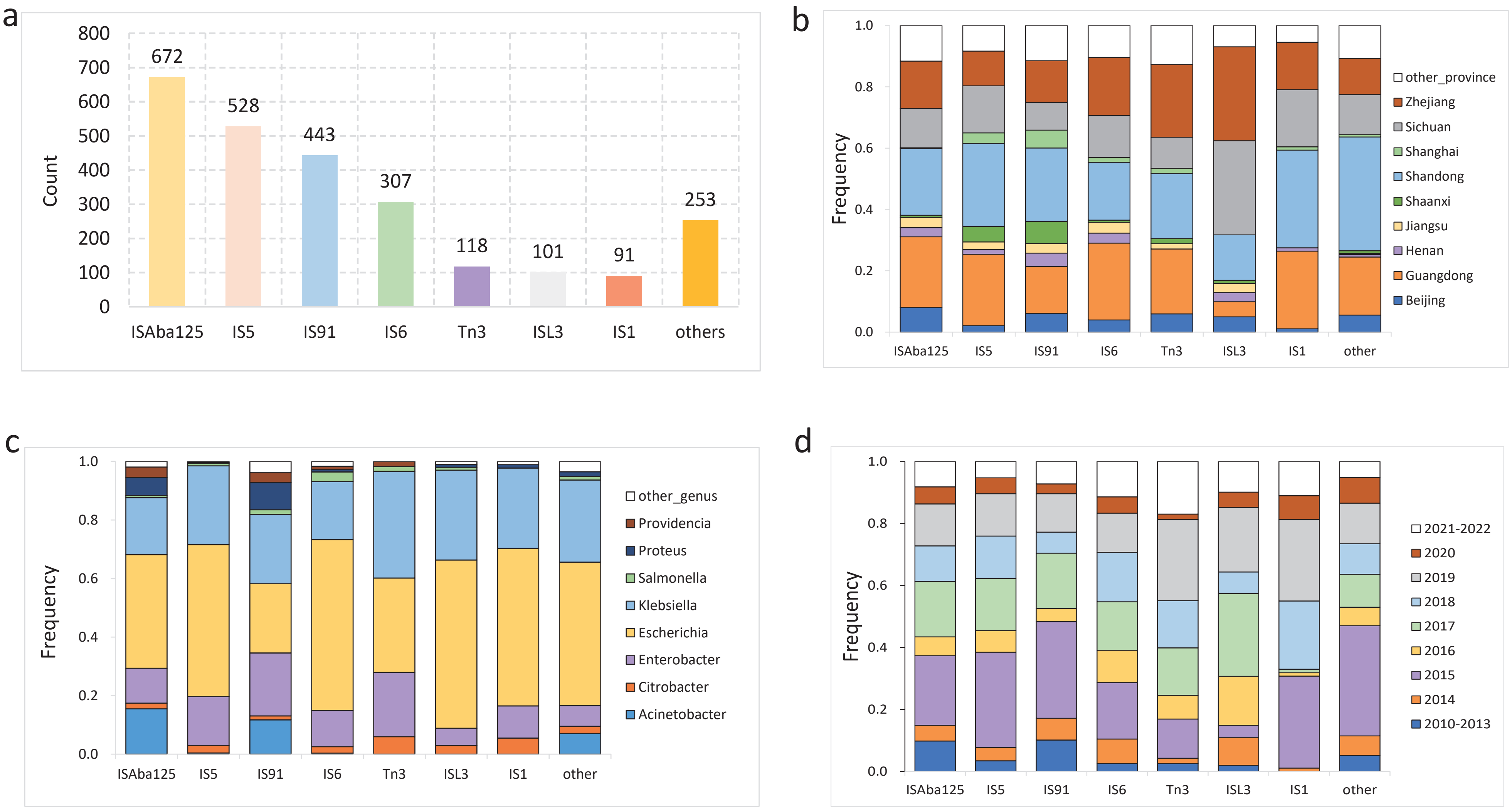

Supplementary Figure 3: Statistical data of the Tn and IS elements upstream and downstream of NDM. a) The number of isolates with the 7 main Tn/IS types as well as other types. The statistic scope included 2 and 9 genes upstream and downstream, from NDM, respectively. **b)** Geographic distribution of the 7 main Tn/IS types as well as other types. Other provinces included Anhui, Gansu, Guangxi, Hainan, Hebei, Hunan, Inner Mongolia, Jiangxi, Jilin, Liaoning, Tianjin, Xinjiang, Yunnan, Hubei, Fujian, and Chongqing. **c)** Distribution of the 7 main Tn/IS types as well as other types at the genus levels. Other genera included Raoultella, Shewanella, Vibrio, Morganella, and Aeromonas. **d)** Distribution of the year of collection of the 7 main Tn/IS types as well as other types.
