# Supplementary material for: Evolution, dissemination, and genetic dynamics of the carbapenem resistance gene bla NDM in China
Source: Front Cell Infect Microbiol. 2025 Aug 11;15:1608826. doi: 10.3389/fcimb.2025.1608826 (PMC12375619; doi:10.3389/fcimb.2025.1608826)
Supplement: Supplementary file 4 [file DataSheet4.pdf]

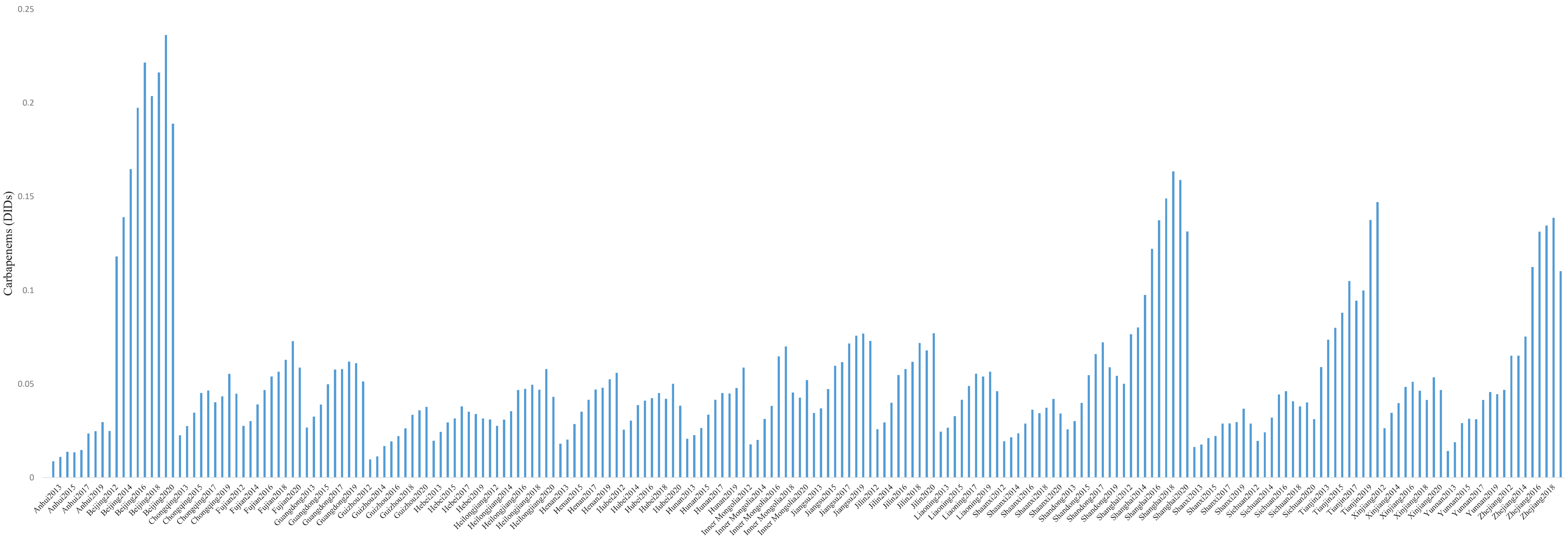

Supplementary Figure 4: The consumption data of antibacterial drugs from 2012 to 2020, which was obtained from the pharmaceutical database of China Pharmaceutical Industry Information Center.
